# Spatial Overlap and Habitat Selection of Corvid Species in European Cities

**DOI:** 10.3390/ani13071192

**Published:** 2023-03-29

**Authors:** Farah Abou Zeid, Federico Morelli, Juan Diego Ibáñez-Álamo, Mario Díaz, Jiří Reif, Jukka Jokimäki, Jukka Suhonen, Marja-Liisa Kaisanlahti-Jokimäki, Gábor Markó, Raphaël Bussière, Marko Mägi, Piotr Tryjanowski, Theodoros Kominos, Antonia Galanaki, Nikos Bukas, Fabio Pruscini, Leszek Jerzak, Olaf Ciebiera, Yanina Benedetti

**Affiliations:** 1 Faculty of Environmental Sciences, Czech University of Life Sciences Prague, Kamýcká 129, 165 00 Prague, Czech Republic; fmorellius@gmail.com (F.M.); ybenedetti73@gmail.com (Y.B.); 2 Institute of Biological Sciences, University of Zielona Góra, Prof. Z. Szafrana St. 1, 65-516 Zielona Góra, Poland; l.jerzak@wnb.uz.zgora.pl (L.J.); o.ciebiera@wnb.uz.zgora.pl (O.C.); 3 Department of Zoology, Faculty of Sciences, University of Granada, 18071 Granada, Spain; jia@ugr.es; 4 Department of Biogeography and Global Change, Museo Nacional de Ciencias Naturales (BGC-MNCN-CSIC), 28006 Madrid, Spain; mario.diaz@mncn.csic.es; 5 Institute for Environmental Studies, Faculty of Science, Charles University, Prague, Benatska 2, 128 01 Praha, Czech Republic; jirireif@natur.cuni.cz; 6 Department of Zoology, Faculty of Science, Palacky University in Olomouc, 17. Listopadu 50, 771 46 Olomouc, Czech Republic; 7 Nature Inventory and EIA-Services, Arctic Centre, University of Lapland, P.O. Box 122, 96101 Rovaniemi, Finland; jukka.jokimaki@ulapland.fi (J.J.); marja-liisa.kaisanlahti@ulapland.fi (M.-L.K.-J.); 8 Department of Biology, University of Turku, 20014 Turku, Finland; juksuh@utu.fi; 9 Department of Plant Pathology, Institute of Plant Protection, Hungarian University of Agriculture and Life Sciences, Ménesi út 44, 1118 Budapest, Hungary; magvacska@gmail.com; 10 4 Route de la Loge, 86800 Liniers, France; raphael.bussiere87@gmail.com; 11 Department of Zoology, Institute of Ecology and Earth Sciences, University of Tartu, 50409 Tartu, Estonia; markomagi78@gmail.com; 12 Institute of Zoology, Poznań University of Life Sciences, Wojska Polskiego 71C, 60-625 Poznań, Poland; piotr.tryjanowski@gmail.com; 13 Department of Zoology, School of Biology, Aristotle University of Thessaloniki, 54124 Thessaloniki, Greece; tkominos@hotmail.com (T.K.); antgalanaki@gmail.com (A.G.); 14 Plegadis, Riga Feraiou 6A, 45444 Ioannina, Greece; bionickbukas@hotmail.com; 15 S. C. della Pantiera 23, 61029 Pantiera, Italy; fabiopruscini@libero.it

**Keywords:** Corvidae, Europe, coexistence, sympatry, urbanization, niche overlap, habitat overlap

## Abstract

**Simple Summary:**

Many corvid species have adapted to live in urban regions. Studying their habitat needs and the similarities among them would allow us to predict species’ responses to global changes. Such studies have not been widely done on generalist species capable of surviving in different environments. Here, we studied the habitat needs and spatial overlap of five corvid species in sixteen European cities. We found significant overlap in the habitats of the corvids, although some had different tendencies. Three species (the Carrion/Hooded Crow, Rook, and Eurasian Magpie) selected open habitats (grass or bare soil). The Eurasian Jay chose more forested areas, and the Western Jackdaw avoided areas with bare soil cover. The species that had similar habitat tendencies also had similar spatial distributions. Our results show that even corvids with different tendencies overlapped highly in their habitats, which means they can tolerate different environmental conditions in urban areas.

**Abstract:**

Understanding habitat and spatial overlap in sympatric species of urban areas would aid in predicting species and community modifications in response to global change. Habitat overlap has been widely investigated for specialist species but neglected for generalists living in urban settings. Many corvid species are generalists and are adapted to urban areas. This work aimed to determine the urban habitat requirements and spatial overlap of five corvid species in sixteen European cities during the breeding season. All five studied corvid species had high overlap in their habitat selection while still having particular tendencies. We found three species, the Carrion/Hooded Crow, Rook, and Eurasian Magpie, selected open habitats. The Western Jackdaw avoided areas with bare soil cover, and the Eurasian Jay chose more forested areas. The species with similar habitat selection also had congruent spatial distributions. Our results indicate that although the corvids had some tendencies regarding habitat selection, as generalists, they still tolerated a wide range of urban habitats, which resulted in high overlap in their habitat niches and spatial distributions.

## 1. Introduction

Although urban areas occupy less than 3% of the total land cover, their impacts reach areas several times larger [1,2]. Additionally, as the urban population is expected to rise to 84% in Europe by 2050, urban land cover is expected to increase even at a greater speed than the population [1,3]. Urban areas are characterized by increased levels of anthropogenic disturbance, noise, light and air pollution, meso-predators (i.e., cats and dogs), and altered environments [4,5,6]. As a result, new environments in which only a few species can survive are created due to urbanization constraints [7], leading to urban communities that are biotically homogenized [8]. Biotic homogenization is characterized by replacing many native, specialist, and endemic species with a few widespread generalists, leading to increasingly similar communities and a reduction in global biodiversity [7,9].

Many corvid species are generalists that adjust to urbanization and anthropogenically modified areas [10,11,12,13]. Most research has reported a positive impact of urbanization on corvids and an increase in their abundance along urbanization gradients [14]. Corvids are intelligent birds with a large brain-to-body mass ratio whose intelligence is comparable to some primates [15]. Their advanced cognition is one of the reasons they are able to thrive amidst urbanization by increasing their innovation to better adapt to new circumstances [15,16]. Corvids may exploit new nesting sites created within artificial structures, such as buildings, poles, tram tracks, and power lines [14,17,18]. Moreover, their omnivore diet allows them to consume different urban foods [15]. Furthermore, decreased predation and persecution pressure in cities are essential factors promoting corvids’ living in urban settings [14]. Due to the low persecution level and anthropogenic food sources in cities, many corvid species have habituated to humans, a factor that further fosters their urbanization [19].

In urban areas, corvids have several impacts on the environment that affect the local people both positively and negatively and, therefore, play a role in the ecosystem services and disservices [20]. Corvids provide several ecosystem services in urban areas as they are seed dispersers of oak and pine trees, could serve as biosensors for the early detection of hazardous contaminating agents (e.g., West Nile Virus), and are considered model organisms of urban ecology studies [14,21,22]. These birds also cause ecosystem disservices as they forage in trash cans, spread waste and possibly diseases, and are known for inducing agricultural and infrastructural damage and causing noise [12,14,23,24]. Corvids are nest predators, and their increased presence in urban areas may limit the nesting capacities of other species, reducing the region’s biodiversity [25,26,27,28,29]. Therefore, detailed knowledge of species’ habitat requirements could contribute to more efficient management of corvids populations in cities when needed [29].

Several studies have looked into the urban habitat selection of corvids [13,30,31,32] and in Europe specifically [18,19,33,34,35]. Most of these studies were only conducted in one city or focused on a single corvid species [19]. Thus, there is still a need for large-scale, meta-replicating studies concerning the urban habitat selection of corvids across species and sites to obtain general findings [36].

Hutchinson defined the realized niche as the environmental conditions where a species can survive, reproduce, and grow despite predators and competitors [37]. The ecological niche governs the distribution of the species and can be considered an n-dimensional hypervolume, where n is the number of ecological factors considered [37,38]. Sympatric species are simultaneously present in the same area [39]. Understanding habitat niche and spatial overlap in guilds of sympatric species in urban areas could aid in predicting both species and community changes in response to global change.

Niche overlap among sympatric birds has been studied extensively in specialist species but neglected in generalist species with broader niches [40]. Some studies have addressed sympatry among corvids outside urban areas [41,42,43,44]. However, studies assessing habitat overlap in corvids in urban areas have been lacking [40].

This study aims to characterize the habitat selection and the degree of spatial overlap among five corvid species inhabiting different European cities. Specifically, we will (1) investigate the presence and distribution of each corvid species in 16 European cities, (2) assess the level of spatial overlap among the five corvid species, (3) understand the level of overlap of their habitat selection, and (4) determine the urban features selected by each species. We hypothesize that due to the differences in body sizes and nest sites, the habitat selection of corvids will differ and that species with similar habitat requirements will have similar spatial distributions. We predict that larger species (the Carrion/Hooded Crow; *Corvus corone/cornix*, Rook; *Corvus frugilegus*, and Eurasian Magpie; *Pica pica*) will pick out more open sites. We believe smaller species (the Eurasian Jay; *Garrulus glandarius*) will choose more vegetated, closed sites. Hole-nesting corvids (the Western Jackdaw; *Corvus monedula*) will select built-up areas. We used the point count method to study the presence and abundance of corvids in the 16 European cities depending on the characteristics of the sites. We then examined the mismatch between their spatial distributions and the level of their habitat niche overlap and modeled the habitat selection of each corvid species.

## 2. Materials and Methods

### 2.1. Study Area

**Figure 1:**
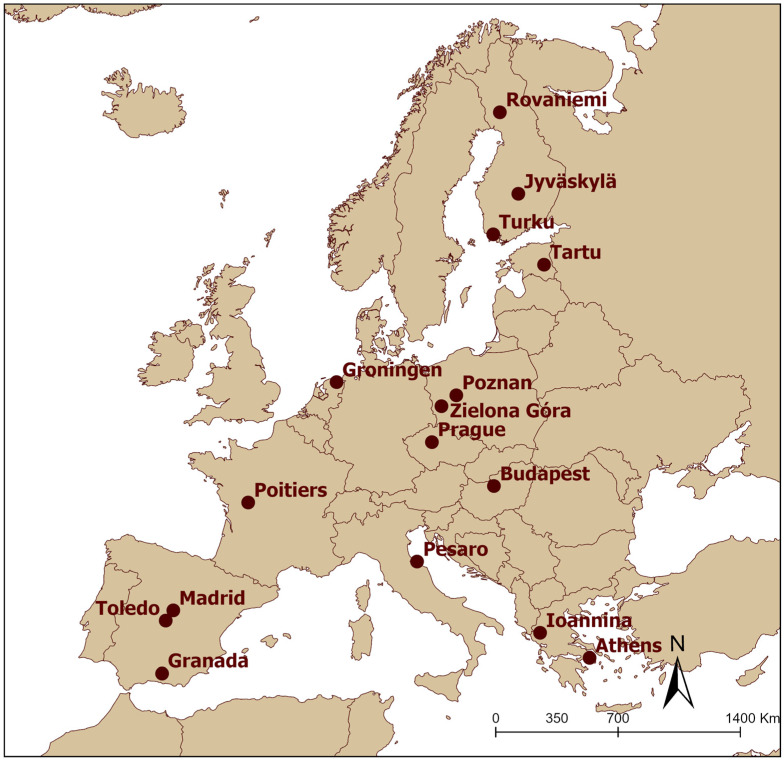
Map of the sixteen European cities surveyed.

In 2018, sixteen European cities (Figure 1) were surveyed during the breeding season. Data on corvid species’ presence and abundance were collected through standardized single-visit point counts [45,46]. We used the point count method to collect data regarding breeding corvids, as we were not interested in roosting corvid flocks that may only overnight in the cities [14]. In each city, around one hundred point counts, with a circle of a 50 m radius, were used for data collection (more information regarding the exact number of point counts in each city, along with the population and population density, is presented in Table S1). All point counts were at least 500 m from the city borders to avoid sampling transitional suburban regions. The distance between any two point counts was more than 100 m to avoid double-counting the same corvid individuals. The point counts were uniformly distributed along a gradient of urbanization (i.e., at the inner core area of the city, the surrounding area of the inner core area, and the less urbanized residential areas) in each city to sample different corvid species with different urban habitat preferences equally.

### 2.2. Field Data Collection

Sampling was started just after sunrise in cities other than Rovaniemi, where the sun does not set during the mid-summer. In Rovaniemi, surveys began at 02.00 a.m. The surveys were ended before the heavy morning traffic started in each study area (i.e., around 07.00 a.m.). Sampling was conducted in favorable weather conditions (no rain or strong wind) and for 5 min per sampling site following standard bird survey methodology used in previous studies on urban birds [45,47,48]. The data were collected during the peak breeding season depending on the city (e.g., May in Southern Spain vs. June in Finland) to maintain a similar detectability of birds between the different cities [49]. The location of each point count was recorded using a GPS to find other characteristics regarding the site (described in the following section). All corvid species and individuals heard or seen within the 50 m radius of the point counts were recorded. Overflying individuals that did not land within the study circle were excluded. 

### 2.3. Variables Studied in Terms of Corvid Habitat Selection

After the bird surveys, information regarding the vegetation cover and land use composition was collected to study the corvids’ habitat characteristics. Local-scale variables were shown to have more influence on the species distribution than regional ones [50]. For that reason, we collected patch-level variables (the percentages of built (impervious), tree (single trees, lines of trees, and tree patches), bush, grass, and bare soil covers; refs. [51,52,53,54], a matrix level variable (the average number of building floors); ref. [55], and disturbance variables (number of cats, dogs, and pedestrians) [6,56]; which were determined visually by the observers within the 50 m radius point count and during the five minutes bird survey period. Other disturbance variables were calculated for each point count. We included light and noise pollution variables while assessing the habitat characteristics of the corvids as these variables have been shown to influence birds’ habitat selection [47,57,58,59].

Each point count was georeferenced. The coordinates of each study circle were used to extract light pollution information from the VIIRS satellite (from the website: https://www.lightpollutionmap.info). The values, precalculated on the website, were extracted for 2018 (here, average yearly values were used) and correspond to the Radiance 10^–9^W/cm^2^ * sr (W = Watts and sr = steradian) [60].

Noise pollution models were performed using the open noise tool (https://plugins.qgis.org/plugins/opeNoise) for QGIS. This tool permits measuring in 2D space (e.g., around point counts) the mean noise from point or road sources received at fixed points and buildings. Noise sources were based on Urban Atlas land use categories, and buildings from Open Street Map (OSM) were used as an advanced input for diffraction and noise reduction. Noise spreading in a 250 m range of each source (point) was calculated. The results consisted of model-based mean noise levels in dB in a radius of 50 m around the point counts [60].

### 2.4. Classification of the Carrion Crow and the Hooded Crow

In 2003, the Hooded Crow was recognized as a separate species from the Carrion Crow due to the positive assortative mating of the two taxa and the reduced fitness of their hybrids [61]. Thus, information regarding each species separately is still lacking and they are often described as both species merged as one [62,63,64,65]. Debate remains regarding the taxonomic classification of the two taxa, where it may seem that they are still in the early stages of speciation [66,67]. From a genetic perspective, their only main difference is possibly their plumage coloration [67,68]. Finally, since both taxa use similar environments in geographically separate areas, estimating their habitat selection and overlap separately may lead to misleading results. For these reasons, we merged the observations of the Carrion Crow and the Hooded Crow and considered them as one species.

### 2.5. Statistical Analyses

To test spatial overlap among the corvid species, we used the spatial mismatch analysis through a Mantel test [69] with the package “ade4” in R [70]. The Mantel test quantifies correlations between two distance matrices using the coefficient RM, which varies between −1 and 1 and behaves similarly to a correlation coefficient. Here, the distance matrices were developed among point counts on the abundance of each corvid species. Monte Carlo permutations, with 999 randomizations, were employed to test for significance.

The nicheROVER package of R [71] was used to estimate the probabilistic niche regions of each species. For each species, point counts where the species was present were used, and then a directional probabilistic niche overlap of each pair of corvid species was deduced for their habitat selection [72]. The niche region is defined as “a 95% probability region in multivariate space”, estimated using 1000 Monte Carlo draws and alpha = 0.95. Niche overlap is then calculated as the posterior probability that an individual from the first species was found within the niche region of the other species and vice versa [72]. The advantages of this approach are that it gives a directional niche overlap metric (overlap of species A into B is different from that of species B into A) and that it accounts for uncertainty using a Bayesian framework. Furthermore, this method is not sensitive to sample size [72]. The latter is particularly useful for calculating overlap among species with different distributions, such as in the case of some pairs of species in our study (i.e., the Western Jackdaw and all other species studied) [73].

Generalized Linear Mixed Models (GLMMs) using a binomial distribution were fitted to assess the characteristics of the habitats used by each corvid species by relating the presence/absence of a corvid species in a point count to the respective predictors. The predictors tested were: the number of cats, dogs, building floors, and pedestrians; the percentage of grass, tree, bare soil, and bush cover; and the amount of light and noise pollution within 50 m around the point counts. The percentage of the built area was dropped for being highly correlated to the percentage of grass cover (VIF > 6). The city (*n* = 14; Rovaniemi and Zielona Góra were excluded from the models for having missing values in the light and noise pollution predictors, making the sample size for the models *n* = 1288) was incorporated as the random factor to account for variation among the different cities. R package “lme4” was used to fit the models [74]. “Dredging” was used from the R package “MuMIn” [75] to form and rank all possible model combinations using the predictors. Second-order Akaike Information Criterion (AICc) was used to select the best models. Model averaging was performed on top models with ΔAICc < 4 (detailed in Supplementary Table S2) to address problems related to selection uncertainty [76] using the MuMIn package.

All analyses were performed using R software version 4.0.3 [77].

## 3. Results

After removing only two observations of the Common Raven (*Corvus corax*), 2324 corvid individuals belonging to five species (the Carrion/Hooded Crow, Rook, Western Jackdaw, Eurasian Jay, and Eurasian Magpie) were recorded in 1462 point counts surveyed in sixteen European cities (Figure 1 and Figure S1).

The Eurasian Magpie was the species most spread in the study area. It was observed in all cities (Figures S2 and S3). The Western Jackdaw was the most abundant corvid and was detected in most cities except Athens, Budapest, and Madrid. The Carrion/Hooded Crow was present in most surveyed cities except Granada, Madrid, and Toledo. The Eurasian Jay was observed in eight cities (Athens, Budapest, Groningen, Ioannina, Poitiers, Poznan, Prague, and Zielona Góra). The Rook was the least detected and least abundant corvid and was only present in five of the sixteen studied cities (Groningen, Poitiers, Poznan, Prague, and Tartu).

### 3.1. Spatial Overlap

The distribution of the Carrion/Hooded Crow was congruent with that of the Rook, Eurasian Jay, and Eurasian Magpie (Table 1). The distribution of the Eurasian Magpie was also slightly congruent with those of the Rook and Eurasian Jay. The spatial distribution of the Western Jackdaw did not match that of any other corvid. The spatial distributions of the Rook and the Eurasian Jay were not congruent. Congruent distributions mean that the species pair had a similar variation in abundance across the point counts.

### 3.2. Habitat Selection

**Figure 2:**
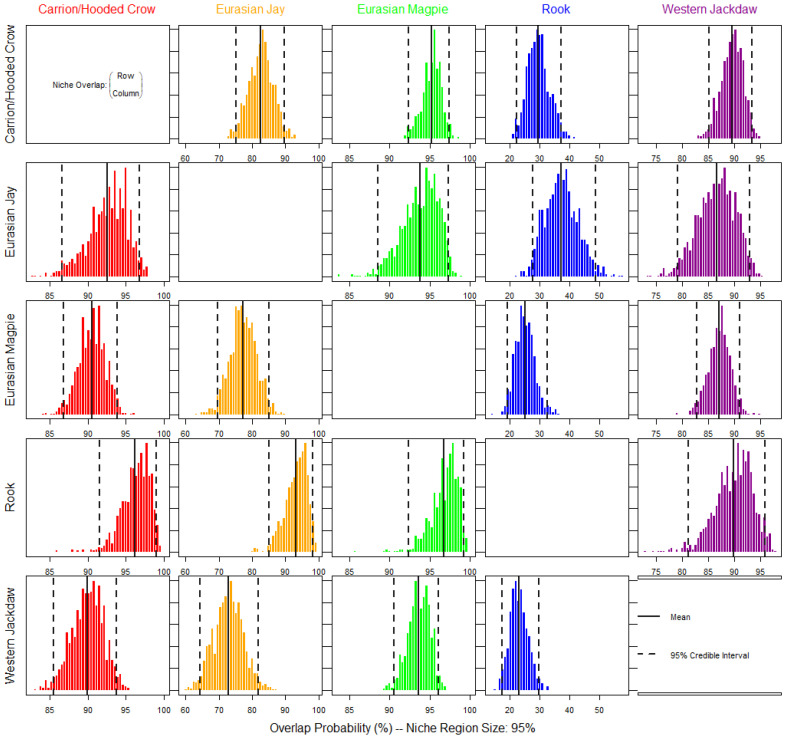
Posterior distribution of the probabilistic niche overlap metric for corvid species in urban areas, considering the land use composition. The posterior mean and 95% credible interval are indicated with black lines and give the probability that species displayed in rows overlap onto those displayed in columns.

The probabilistic niche overlap between two species is not necessarily identical. This approach gives a directional niche overlap metric (overlap of species A into B is different from that of species B into A) [72]. The overlap of habitat use was high between each pair of corvids, with the probability of overlap of all pairs being higher than 80%, except for the Rook (Figure 2). The probability that any corvid individual overlaps the habitat niche region of the Rook was below 40%. On the other hand, the probability that a Rook individual would overlap the habitat niche region of any other corvid was higher than 85%.

Specifically, the percent cover of bare soil and grass were the two most important variables to characterize the habitat use of all corvid species (Table 2). The Carrion/Hooded Crow presence was positively correlated to bare soil and also to grass cover as the Rook. The presence of the Western Jackdaw was negatively correlated to the cover of bare soil. The Eurasian Jay’s presence was positively correlated to the percentage of tree cover. The Eurasian Magpie’s presence positively correlated to bare soil, grass cover, and noise level. However, it was negatively correlated to the number of pedestrians present. No corvid species’ presence was significantly correlated to the number of cats, dogs, building floors or the amount of light within the 50 m radius.

## 4. Discussion

### 4.1. Corvids’ Abundance and Distribution in Urban Areas

The Eurasian Magpie was the most widespread species and present in all sixteen surveyed European cities (Figures S1 and S2). The species started colonizing Eurasian cities during the second half of the twentieth century [34,78,79]. Magpies are omnivorous and sedentary, traits facilitating a bird’s presence in urban environments [80]. Eurasian Magpies could modify their behavior to adapt to urban areas and have already undergone synurbanization in several cities [31,33,34,35,81]. For example, the bird tends to nest higher in trees as urbanization levels increase [32,33,35]. In urban areas, the Eurasian Magpie increases the share of the nests it builds in conifers, especially in early spring when deciduous trees are leafless and exposed [33,34]. These adaptations probably allow Eurasian Magpie individuals to avoid human disturbance and nest predation from pets and Carrion Crows [32,33,34]. The decrease in persecution is another apparent reason for the urbanization of the Eurasian Magpie [34].

The *Corvus* genus is an especially successful genus within the Corvidae family. Their successful global expansion (as they occupy all continents but Antarctica) is due to their capacity to disperse over long distances and their high ability to survive in suboptimal and adapt to new environments [10]. The Western Jackdaw was the most abundant species (Figures S1 and S2). As a cavity nester, the species has adapted to use buildings and other anthropogenic cavities for nesting in urban areas [11,17,82,83]. In Slovenia, a study found that more than 80% of Western Jackdaw pairs nest in buildings [17]. The Western Jackdaw is also an omnivore and somewhat sedentary, so adaptation to urbanization is no surprise [84].

The Carrion/Hooded Crow was also abundant and widespread (Figures S1 and S2). The Carrion/Hooded Crow has also been frequently associated with urbanization, anthropogenically modified areas, and anthropogenic food resources [12]. The Carrion/Hooded Crow has benefitted from the decreased persecution [85]. The Carrion/Hooded Crow, Western Jackdaw, and Eurasian Magpie were the three most common corvids observed in a study conducted during the winter season in urban areas of Finland [19].

Our study, conducted during the breeding season, showed that the Eurasian Jay and Rook were the least spread and abundant species (Figures S1 and S2). The densities of the Rook are declining in Europe, and the bird has been listed as vulnerable on the European Red List of Birds [86]. Additionally, the Rook and Eurasian Jay have been previously demonstrated to utilize cities more often during the winter, perhaps to use warmer temperatures and ample food supplies. For the breeding season, both species probably move to nearby villages to nest and feed in more natural areas, which explains the low number of their records in our sample [11,87,88,89]. In addition, the Eurasian Jay has not yet become urbanized in some regions, such as Finland [19].

### 4.2. Corvids’ Urban Habitat Selection

Understanding the habitat requirements of corvids in urban areas could aid efforts to control their populations and reduce their negative impacts [14,29]. Our large-scale study investigated the urban habitat of five corvid species in sixteen European cities during the breeding season. Although some species showed different habitat selections, the majority (the Carrion/Hooded Crow, Rook, and Eurasian Magpie) were positively linked to open spaces (grass and bare soil cover; Table 2). This selection may be related to their feeding habits because various studies have shown that the abundance and habitat selection of corvids in cities were influenced by food availability [12,24,40]. Although they use anthropogenic food sources such as waste disposal sites, they also rely on insects, snails, and earthworms, especially during the breeding season, to provide their juveniles with nutritious food [12,15,84,90]. Therefore, their presence increases near open grass and bare soil fields where they could be foraging for these valuable resources. Another advantage of open habitats is the early detection of predators since few structures obscure their vision [91]. As corvids are relatively heavy birds, they require longer to flee from approaching predators, so early detection of predators may be valuable [92]. Other studies also found a positive correlation between open spaces and corvids within and outside of urban areas, especially grasslands [18,89,93,94,95]. The Western Jackdaw was the only corvid negatively impacted by bare soil cover (Table 2). Here, the percentage of the built surface was dropped from the models as it was highly and negatively correlated to the portion of grass and, to a lesser extent, bare soil. More extensive coverage of bare soil would translate to a smaller cover of built-up areas. Unlike the other corvids in this study, the Western Jackdaw, as a cavity nester, is known to nest in buildings [17,32,82,83], which could explain its negative correlation to bare soil cover, resulting from reduced built-up areas and, thus, nesting sites. Outside of urban areas, the Western Jackdaws are found in farmlands, rocky habitats, or a mix of both, where they can have nesting and feeding sites [83]. Other studies found a positive correlation between the Western Jackdaw abundance and city centers or densely built-up areas [52,83,96]. Within urban areas, Salvati (2002), found that the optimal habitat of the Western Jackdaw consists of a mixture of old buildings, ruderal zones, open areas, and small green areas [83]. From our results, it seems that the built cover is the most important of these factors and that Western Jackdaws may choose regions with more extensive built cover and smaller open land covers for breeding. There might even be a mismatch between the nest sites of urban Western Jackdaw individuals and their optimal foraging habitats [84], which suggests regular movements of the species between nesting sites (in cities) and foraging sites (their surroundings), and hence an effect of city size on the Western Jackdaw’s presence. The Eurasian Jay was not linked to the cover of the open areas (Table 2). It was the only corvid in this study positively influenced by tree cover. It is not surprising as the Eurasian Jay has been considered a typical forest dweller associated with forest cover [11,97], is still in the process of colonizing urban areas [93,98], and is more correlated to the least urbanized sectors of a city [99]. Moreover, the Eurasian Jay may actively increase the tree cover of a city because this species is considered an efficient disperser of acorn through a mutualistic relationship with oak species [97,100,101]. Another study showed a positive correlation between the Eurasian Jay and woody vegetation in an urban area, matching our findings [93]. None of the corvids studied seemed to be impacted by the amount of light (Table 2). These results differ from those of another study that found that the densities of the Rook and Eurasian Magpie increased with light pollution levels and decreased with the noise level in southern Poland [58]. The different spatial scales and the fact that the former study was conducted during the winter season may explain these differences. In contrast, our results only showed a positive relationship between noise pollution and the presence of the Eurasian Magpie and no impact on other corvids (Table 2). Some species may benefit from higher noise levels due to the disruption of predator–prey interactions, which may be the case of the Eurasian Magpie [59]. The Eurasian Magpie was also the most widespread corvid in our study. Both results suggest that the Eurasian Magpie is a flexible corvid and the most tolerant to urban noise pollution in the European cities studied. Still, all corvid species studied are well adapted to urban noise and not heavily impacted by it. Similarly, another study found that the Eurasian Magpie and Western Jackdaw were linked to areas with increased noise levels [52]. The Eurasian Magpie was also the only corvid impacted by the density of pedestrians. The amounts of cats and dogs affected none of the corvids studied. Although these mesopredators may be more abundant in urban areas, predation rates are lower as they may be relying on anthropogenically abundant food, shifting their diets away from vertebrate prey, something corvids may have caught up with [6,102].

### 4.3. Corvids’ Spatial and Habitat Overlap

The habitat selection of the corvids could explain their spatial distributions and level of habitat overlap. The distribution of the Western Jackdaw was not congruent with any other corvid (Table 1). We assume this is due to the Western Jackdaw’s preference for built-up and heavily dense areas [96], unlike the other corvids. The Carrion/Hooded Crow, Rook, and Eurasian Magpie were linked to open spaces, and their distributions were congruent (Table 1 and Table 2). The Eurasian Jay was the only one related to the tree cover, unlike other corvids, its distributions matched those of the Eurasian Magpie and Carrion/Hooded Crow. This could be due to their occurrence in large urban open spaces, such as parks, where large open spaces and tree covers coincide, benefiting both species similarly. The distribution of the Eurasian Jay was not congruent with that of the Rook. We assume this is caused by the low presence of both species in this study.

We found a high overlap in this study’s habitat niches of all five corvid species (Figure 2). High habitat overlap of four corvids (the Carrion/Hooded Crow, Rook, Western Jackdaw, and Eurasian Magpie) was also found in winter in agricultural areas in Britain [95]. Here, except for the Rook, the probability that any corvid overlapped another corvid’s habitat niche region was very high [95]. The probability that another corvid overlapped the niche of the Rook was low but high the other way around. This indicates that the Rook has a smaller niche region, almost completely embedded in the different corvids’ niches. We expect Rooks to broaden their urban habitat niches during the winter when they are more likely to occupy this environment [96]. The Eurasian Magpie, followed by the Carrion/Hooded Crow, had the largest niches that highly overlap and almost embed to a large extent within them the majority of the habitat niches of other species. The Eurasian Magpie also had the largest overlap in foraging behavior with other corvids in another study [41]. As for the Western Jackdaw and Eurasian Jay, although they highly overlapped, they had the least habitat niche overlap between them, perhaps because the Eurasian Jay selected more natural areas [98], and was correlated to tree cover, while the Western Jackdaw may select built-up areas [83,96].

While some corvids seemed to select similar habitats in urban areas (the Eurasian Magpie, Carrion/Hooded Crow, and Rook), others had different tendencies (the Western Jackdaw and Eurasian Jay), they still overlapped quite extensively in their habitat niches (Table 1, Figure 2). In addition, although their niches highly overlapped, the corvids distributions were congruent only with those with similar tendencies. We can infer that corvids can tolerate a wide array of ecological conditions in urban regions but still have some preferences [14]. They are intelligent birds with an omnivore diet which aids them in broadening their ecological niches by adapting to novel environments and using different foods [12,15,16]. Thus, behavioral adaptations might play an essential role in adapting species to novel environments, especially in unstable or disturbed ones [10]. The high habitat overlap paired with increased congruent distributions between species of similar habitat selection could also be explained by the fact that birds have only started to colonize urban areas recently. Their urban populations may not have yet reached the carrying capacity and resource limitations of the environment, and thus, the pressure upon those closely related species that need to acquire interspecific differentiation may be still too weak, enabling their coexistence even in the presence of broad niche overlap [103,104]. In addition, high disturbance regimes, such as urban areas, tend to allow the coexistence of generalists with overlapping niches [105]. Alternatively, since their habitats overlap largely, we think other factors, unaccounted for in this study, may determine separation in their resource use. For example, while four corvids highly overlapped in their foraging habitats, their overlap in their prey type intakes was low [41]. Moreover, vast morphological differences in the skulls of corvids were noted, which were attributed to their differences in foraging modes [106]. In an urban study, two sympatric crow species were found to differ in feeding behaviors and feeding habitat, while their food preferences overlapped extensively [40]. Thus, interspecific relationships (i.e., territoriality and dominance) may also impact their use of shared resources. Corvids may demonstrate aggressive behavior against other species when foraging if the overlap is high or avoid an area if another species is feeding [41]. Corvids also change their feeding preferences in larger flocks, indicating that interspecific relationships may impact resource use [107]. A study assessed four corvid species’ segregation in using a refuse dump and found temporal (daily and seasonal) differentiation in its use by the different corvids [42]. Kleptoparasitism by the Carrion Crow against the other corvids was noted, which may have contributed to the temporal segregation in using this shared resource [42].

Since our study was conducted during the breeding season only, and some corvids (i.e., the Rook and Eurasian Jay) were shown to utilize urban areas more often during the winter, we expect different levels of habitat niche overlap among the corvids during the winter season, especially as wintering birds were shown to be more generalist in their habitats than breeding birds [11,19,87,88,89]. Many corvids were previously found to use urban areas for nocturnal roosting [14,17]. Our data collection was only conducted in the morning and focused on breeding corvids but future research could investigate corvid habitat use at different times of the day. Furthermore, other corvids that may be urbanized in Europe have not been reported in our study (i.e., the Common Raven of which we only had two observations that were then dropped) [19]. Thus, other factors than the conditions of our study may determine their presence [19].

## 5. Conclusions

We studied the distribution, habitat selection, and spatial and habitat niche overlap of five corvid species in sixteen European cities during the breeding season. We found that three corvids were quite spread and abundant (The Carrion/Hooded Crow, Western Jackdaw, and Eurasian Magpie), while two were less present (the Rook and Eurasian Jay). High habitat overlap has been observed among the five studied corvids. Although their habitats highly overlapped, the species still had some tendencies in their habitat selection. Three corvid species selected urban areas with open spaces (the Carrion/Hooded Crow, Rook, and Eurasian Magpie). The Eurasian Jay was linked to increased tree cover. The Western Jackdaw was negatively correlated to bare soil cover. Species with similar habitat selection had congruent distribution. Our results are not surprising since corvids are highly adaptable generalists expected to have broad niches and, therefore, overlap in their habitats and spaces [14,29]. We assume that other factors, to be investigated in future studies, may impact their sympatric relationships, habitat, and spatial overlap, such as the season, time of day, interspecific interactions, and dietary preferences and habits [40,41,42,95,107].

## Figures and Tables

**Table 1:** Spatial overlap test. Results of Mantel tests between the spatial distributions of each pair of corvid species, with 999 Monte Carlo permutations. The table shows the statistic RM of the test and the simulated *p*-values. Values with a *p*-value < 0.05 are in bold.

Variables Correlated	RM	*p*-Value
**Carrion/Hooded Crow × Rook**	**0.100**	**<0.01**
Carrion/Hooded Crow × Western Jackdaw	−0.010	>0.05
**Carrion/Hooded Crow × Eurasian Jay**	**0.110**	**<0.01**
**Carrion/Hooded Crow × Eurasian Magpie**	**0.150**	**<0.001**
Rook × Western Jackdaw	0.035	>0.05
Rook × Eurasian Jay	−0.016	>0.05
**Rook × Eurasian Magpie**	**0.084**	**<0.01**
Western Jackdaw × Eurasian Jay	−0.002	>0.05
Western Jackdaw × Eurasian Magpie	0.010	>0.05
**Eurasian Jay × Eurasian Magpie**	**0.080**	**<0.01**

**Table 2:** Habitat selection models. Results of the model averaged coefficients of the GLMMs relating corvid species’ presence/absence to urban habitat characteristics. The predictors include the noise and light pollution, the percentage of bare soil, grass, tree, and bush cover, and the number of pedestrians, cats, and dogs present in the 50 m radius around the point counts. The city (*n =* 14) was used as a random factor. A separate model was run for each corvid species. For each species, dredging was used to generate all models based on the various combinations of predictors. Models with ∆AICc < 4 (top models are detailed in Supplementary Table S2.) were averaged to give the results in the table. Estimates with a *p*-value < 0.05 are in bold.

Variable	Estimate	SE	z-Value	*p*-Value
Carrion/Hooded Crow				
Intercept	−3.050	0.737	4.141	*p* < 0.001
**Bare soil**	**0.022**	0.007	2.977	**0.003**
Building floors	0.068	0.048	1.421	0.155
Bush	0.002	0.008	0.192	0.848
Cats	−0.019	0.083	0.224	0.823
Dogs	0.118	0.074	1.595	0.111
**Grass**	**0.016**	0.005	3.200	**0.001**
Light	−0.000	0.004	0.058	0.954
Noise	−0.007	0.011	0.587	0.557
Pedestrians	−0.008	0.004	1.756	0.079
Tree	−0.000	0.006	0.069	0.945
Rook				
Intercept	−5.551	1.952	2.843	0.004
Bare soil	0.019	0.017	1.115	0.265
Building floors	−0.173	0.139	1.246	0.213
Bush	−0.044	0.026	1.706	0.088
Cats	−0.012	0.292	0.041	0.968
Dogs	−0.390	0.291	1.343	0.179
**Grass**	**0.034**	0.011	2.947	**0.003**
Light	−0.016	0.016	0.990	0.322
Noise	0.004	0.031	0.139	0.890
Pedestrians	−0.010	0.019	0.512	0.609
Tree	−0.034	0.018	1.923	0.055
Western Jackdaw				
Intercept	−1.605	0.7629	2.104	0.035
**Bare soil**	**−0.016**	0.0070	2.350	**0.019**
Building floors	0.053	0.038	1.374	0.170
Bush	−0.008	0.008	0.922	0.357
Cats	0.043	0.086	0.498	0.619
Dogs	−0.140	0.084	1.665	0.096
Grass	−0.009	0.005	1.736	0.083
Light	0.004	0.003	1.304	0.192
Noise	−0.009	0.011	0.800	0.424
Pedestrians	0.000	0.002	0.198	0.843
Tree	−0.010	0.006	1.557	0.120
Eurasian Jay				
Intercept	−4.795	1.551	3.092	0.002
Bare soil	0.005	0.012	0.419	0.675
Building floors	−0.238	0.135	1.766	0.077
Bush	0.017	0.013	1.331	0.183
Cats	0.131	0.105	1.250	0.211
Dogs	0.089	0.112	0.796	0.426
Grass	0.010	0.010	1.041	0.298
Light	−0.012	0.009	1.322	0.186
Noise	0.026	0.021	1.237	0.216
Pedestrians	−0.027	0.015	1.767	0.077
**Tree**	**0.019**	0.009	2.186	**0.029**
Eurasian Magpie				
Intercept	−2.914	0.695	4.191	<0.001
**Bare soil**	**0.015**	0.006	2.776	**0.006**
Building floors	0.021	0.030	0.709	0.478
Bush	0.002	0.007	0.245	0.806
Cats	0.004	0.075	0.053	0.957
Dogs	−0.025	0.062	0.401	0.689
**Grass**	**0.027**	0.004	6.644	**<0.001**
Light	−0.003	0.003	1.151	0.250
**Noise**	**0.021**	0.009	2.359	**0.018**
**Pedestrians**	**−0.012**	0.004	3.385	**<0.001**
Tree	−0.003	0.005	0.645	0.519

## Data Availability

The authors can provide additional information regarding the raw data directly, under reasonable request.

## Supplementary Materials

The following supporting information can be downloaded at: https://www.mdpi.com/article/10.3390/ani13071192/s1; Figure S1: The distribution of the different corvid species in the surveyed cities; Figure S2: Proportion of detections of each corvid species at point counts and cities; Figure S3: Frequency of point counts with a certain corvid abundance in each of the surveyed cities; Table S1: The surveyed cities along with their latitudes, longitudes, populations, population densities (/km^2^) and the number of point counts in each.; Table S2: Top models (defined by ∆AICc < 4) describing the relationships between the presence/absence of the corvid species and the predictors.
