# Supplementary material for: Spatial Overlap and Habitat Selection of Corvid Species in European Cities
Source: Animals (Basel). 2023 Mar 29;13(7):1192. doi: 10.3390/ani13071192 (PMC10093487; doi:10.3390/ani13071192)
Supplement: Supplementary file 1 [file animals-13-01192-s001.zip › Supplementary_Material_Animals-2288877.pdf]

SUPPLEMENTARY MATERIAL

Table S1. The surveyed cities along with their latitudes, longitudes, populations, population densities (/km^2) and the number of point counts in each. When available, the population densities of the core area of the cities were also included (inside parentheses). The population and population densities were obtained from the respective Wikipedia pages of each city.

Country	City	Latitude	Longitude	Population	Population density (/km^2)	Sample size
Czech Republic	Prague	50.07346	14.49901	1,275,406	2,570	119
Estonia	Tartu	58.3836	26.72062	91,407	2412	99
Finland	Jyväskylä	62.243	25.7617	144,447 (117,974)	123 (1,189)	89
	Rovaniemi	66.503059	25.726967	64,194 (53,361)	8 (904)	83
	Turku	60.45	22.254	195,301	795	85
France	Poitiers	46.56191	0.312026	89,212	2,119	100
Greece	Athens	37.97294	23.7334	3,059,764	7427	96
	Ioannina	39.65587	20.851312	112,486 (80,371)	279 (2371)	93
Hungary	Budapest	47.47956	19.04851	1,752,286	3,336	86
Italy	Pesaro	43.912476	12.915549	95,000	749	55
Netherlands	Groningen	53.219383	53.219383	233,218	1,256	99
Poland	Poznan	52.41151	16.817517	529,410	2,022	100
	Zielona Góra	51.90575	15.507097	140,403	510	60
Spain	Granada	37.19989	-3.629306	232,208	2,638	99
	Madrid	40.44021	-3.687409	3,223,334	5,334	99
	Toledo	39.8614	-4.042886	84,282	360	100
			Total sample size			1462

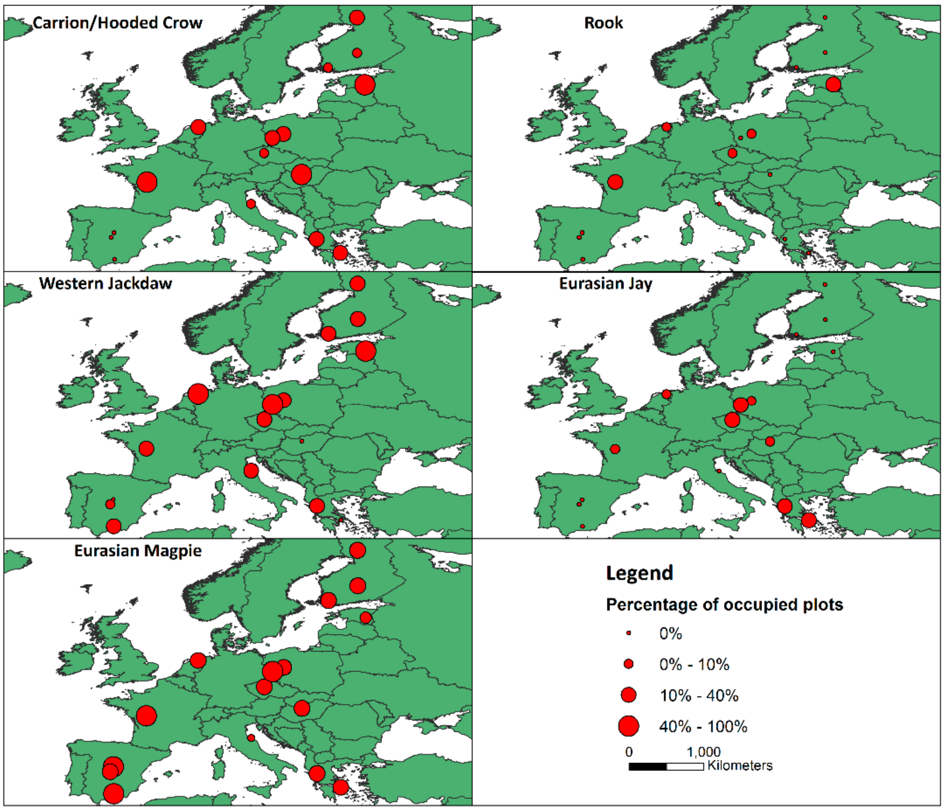

Figure S1. The distribution of the different corvid species in the surveyed cities. The size of the circles indicates the percentage of occupied point counts in each city.

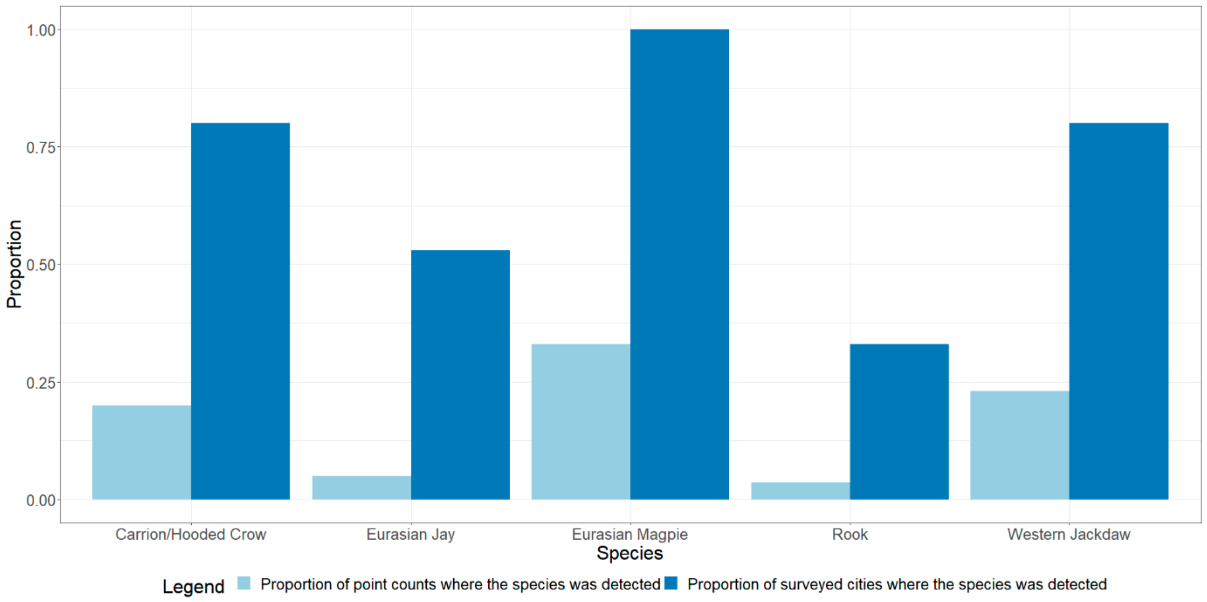

Figure S2. Proportion of detections of each corvid species at point counts (light blue bars) and cities (dark blue bars).

Figure S3. Frequency of point counts with a certain corvid abundance in each of the surveyed cities.

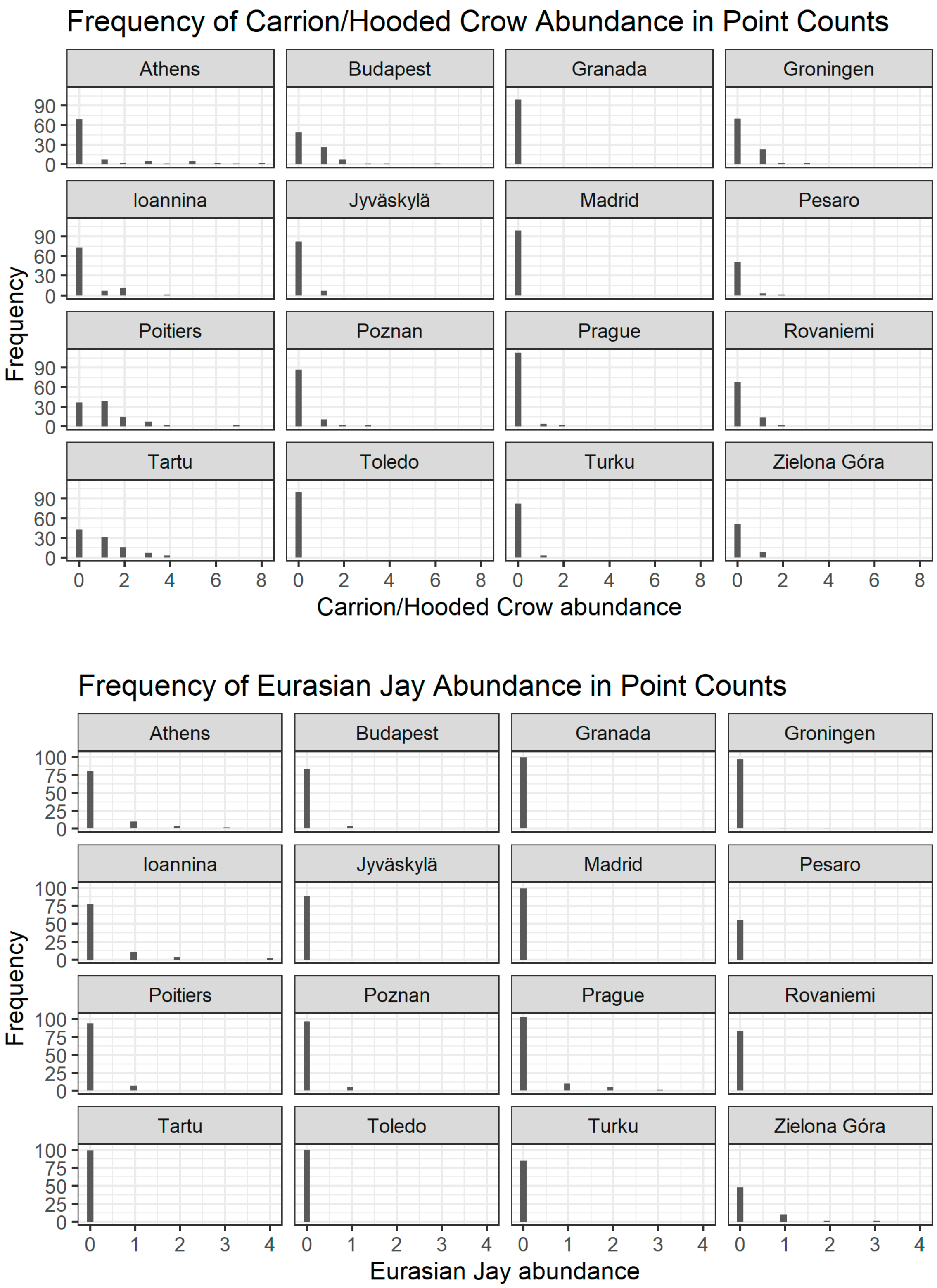

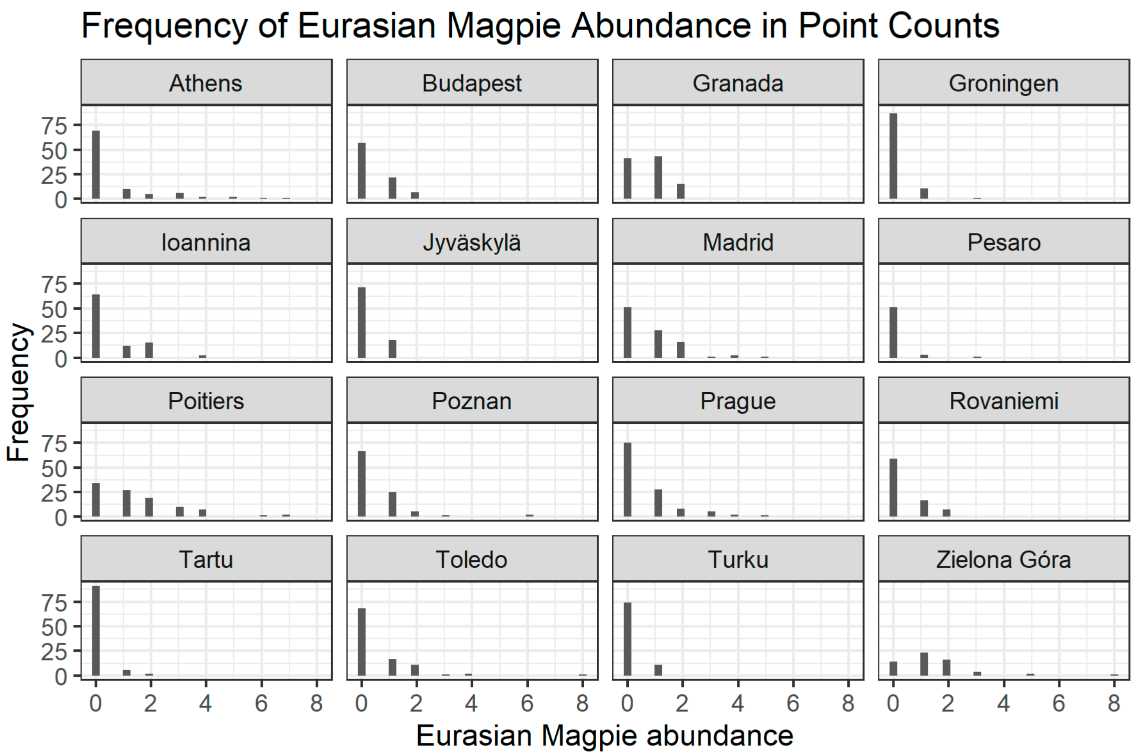
Frequency of Eurasian Magpie Abundance in Point Counts
Athens
Budapest
Granada
Groningen
Ioannina
Jyväskylä
Madrid
Pesaro
Poitiers
Poznan
Prague
Rovaniemi
Tartu
Toledo
Turku
Zielona Góra
Frequency
0
25
50
75
0
2
4
6
8
Eurasian Magpie abundance

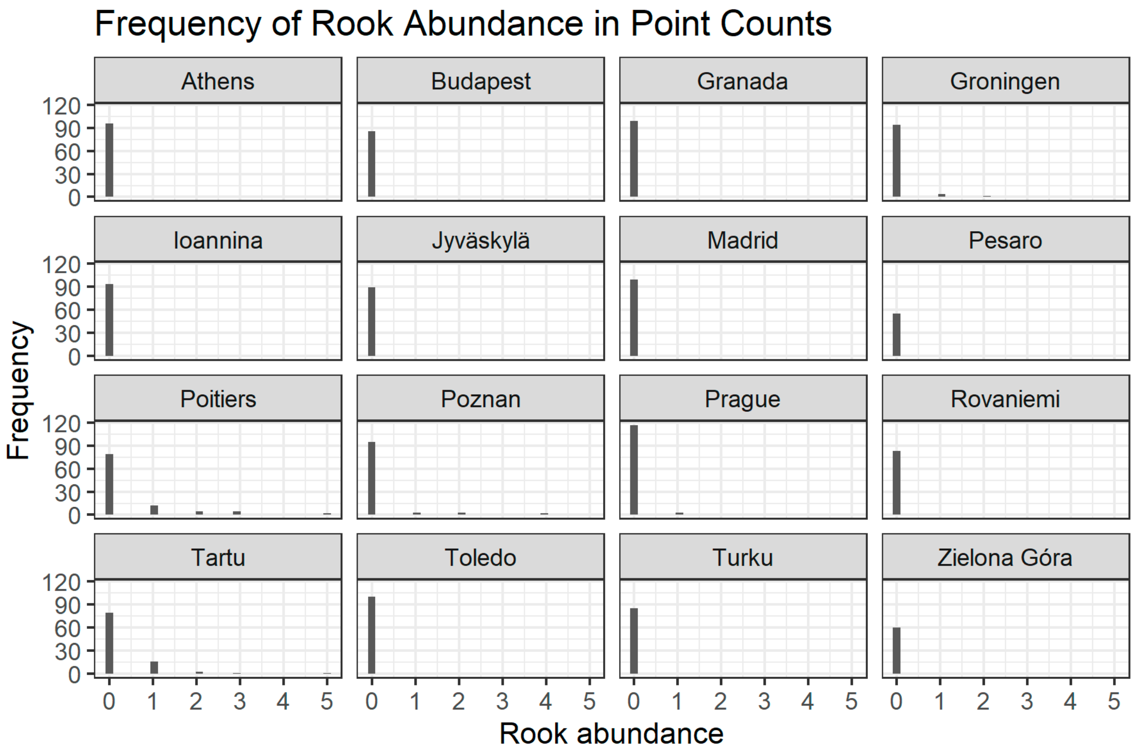
Frequency of Rook Abundance in Point Counts
Athens
Budapest
Granada
Groningen
Ioannina
Jyväskylä
Madrid
Pesaro
Poitiers
Poznan
Prague
Rovaniemi
Tartu
Toledo
Turku
Zielona Góra
Frequency
0
30
60
90
120
0
1
2
3
4
5
Rook abundance

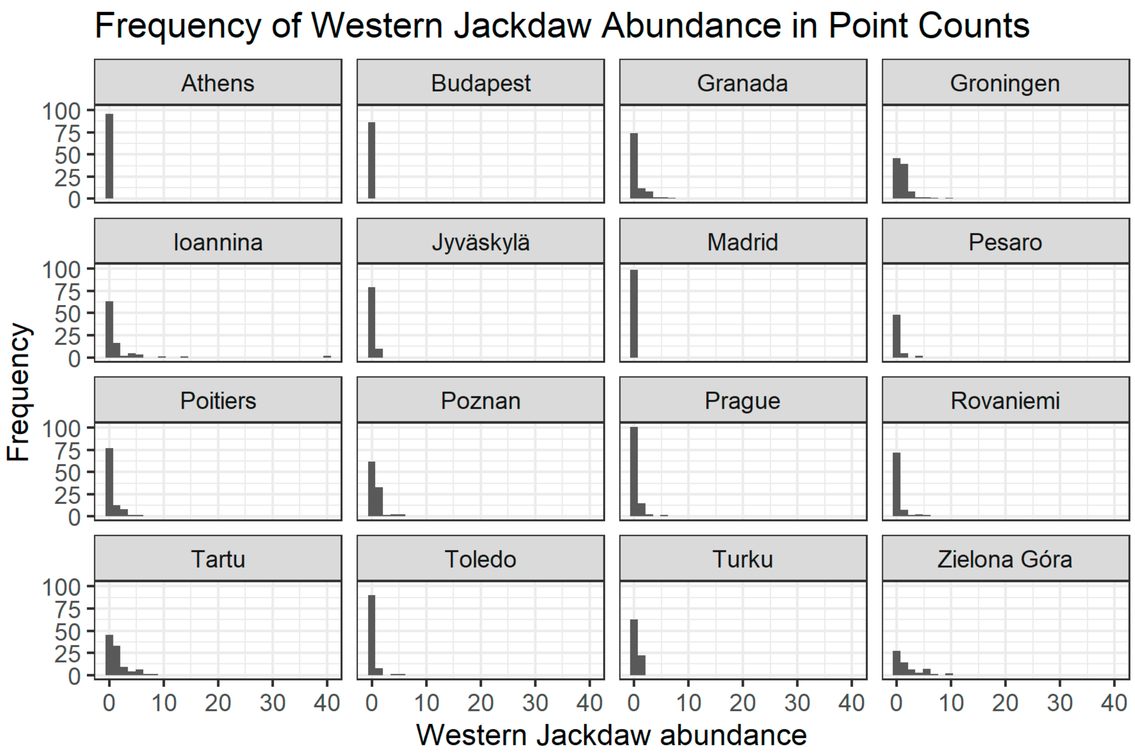

Frequency of Western Jackdaw Abundance in Point Counts
Athens
Budapest
Granada
Groningen
Ioannina
Jyväskylä
Madrid
Pesaro
Poitiers
Poznan
Prague
Rovaniemi
Tartu
Toledo
Turku
Zielona Góra
Frequency
100
75
50
25
0
0
10
20
30
40
Western Jackdaw abundance
